# Study on influencing factors of anxiety symptoms and its association with nutritional condition in elderly heart failure patients

**DOI:** 10.3389/fnut.2026.1861582

**Published:** 2026-08-12

**Authors:** Qiao Hu, Ting Yao, Miaomiao Li, Yukuan Miao, Zheyuan Xia, Xiang Wang

**Affiliations:** 1 The School of Nursing, Anhui University of Chinese Medicine, Hefei, China; 2 Laboratory of Geriatric Nursing and Health, Anhui University of Chinese Medicine, Hefei, China; 3 Emergency Intensive Care Unit (EICU), The First Affiliated Hospital of Anhui Medical University, Hefei, China

**Keywords:** anxiety, elderly heart failure, influencing factors, nutritional condition, quality of life

## Abstract

**Objective:**

To analyze influencing factors of anxiety symptoms in elderly heart failure patients and explore interrelationships between nutritional status, anxiety, and heart failure severity, to provide evidence for the formulation of integrated clinical intervention schemes combining psychological care and nutritional support.

**Methods:**

This cross-sectional study included 204 elderly heart failure patients (age ≥65 years) hospitalized at The First Affiliated Hospital of Anhui University of Chinese Medicine’s cardiovascular medicine department between May and December 2025. Patients’ anxiety status was assessed using the Self-Rating Anxiety Scale (SAS) and categorized into an Anxiety group and a non-Anxiety group accordingly. Patients’ socio-demographic data, clinical information, and laboratory indicators including N-terminal pro-brain natriuretic peptide (NT-proBNP), total protein (TP), and albumin (ALB) were collected; Nutritional condition, family support, and quality of life were concurrently assessed using the Mini Nutritional Assessment Short-Form (MNA-SF), Family Care Index Questionnaire (APGAR), and Minnesota Living with Heart Failure Questionnaire (MLHFQ). Binary logistic regression analysis was performed to identify independent influencing factors for anxiety; Spearman’s rank correlation analysis was used to assess correlations between nutritional status and clinical indicators; and multiple linear regression analysis was conducted to identify independent influencing factors for nutritional status.

**Results:**

Among 204 patients, 48.5% (*n* = 99) experienced anxiety. Significant differences were observed between the Anxiety group and the non-Anxiety group in NYHA cardiac function classification, number of comorbid chronic cardiovascular diseases, living alone status, NT-proBNP, HDL-C, LDL-C, TP, ALB, nutritional status, family care index, and quality of life (all *p* < 0.05). Binary logistic regression analysis revealed that NYHA cardiac function classification (OR = 7.826), NT-proBNP (OR = 12.177), and LDL-C (OR = 2.029) were independent risk factors for anxiety in elderly patients with heart failure (all *p* < 0.05), while HDL-C (OR = 0.264) and MNA-SF total score (OR = 0.434) were independent protective factors (all *p* < 0.05). Spearman’s rank correlation analysis demonstrated that the MNA-SF total score was significantly negatively correlated with NYHA cardiac function classification (rs = −0.36), number of comorbid chronic cardiovascular diseases (rs = −0.324), NT-proBNP (rs = −0.334), LDL-C (rs = −0.163), MLHFQ total score (rs = −0.62), and SAS total score (rs = −0.832), while showing significant positive correlations with HDL-C (rs = 0.139), TP (rs = 0.276), ALB (rs = 0.258), and APGAR total score (rs = 0.402) (all *p* < 0.05). Multiple linear regression analysis revealed that higher APGAR total scores were associated with better nutritional status in patients; whereas elevated NT-proBNP levels, MLHFQ total scores, and SAS total scores were correlated with poorer nutritional status (all *p* < 0.05).

**Conclusion:**

The prevalence of anxiety symptoms was high among elderly heart failure patients. Anxiety levels were significantly correlated with worse NYHA grading, elevated NT-proBNP and LDL-C, and poorer nutritional status. There was a strong negative correlation between anxiety and nutritional status, and these three indicators were simultaneously linked to unfavourable heart failure progression. Based on the observed correlational relationships, integrated psychological and nutritional assessment and intervention are recommended for elderly patients with heart failure in clinical practice. This cross-sectional study can only identify correlational associations rather than causal relationships; prospective cohort studies are required to clarify the directional interactions among heart failure severity, anxiety and nutritional status.

## 1 Introduction

Heart Failure (HF), commonly abbreviated as HF, is a chronic progressive clinical syndrome caused by structural or functional abnormalities of the heart. Characterized by complex etiology and diverse pathophysiological mechanisms, it has become a major global public health challenge (1, 2). With the accelerating aging of the population, heart failure is particularly prevalent among the elderly. The decline in physiological function, geriatric syndromes, and multimorbidity further increase vulnerability to the disease in this population (3). Epidemiological data indicate that the prevalence rates among Chinese populations aged 65–79 years and ≥80 years reach 3.86 and 7.55%, respectively (4). This disease not only has a high incidence rate but also imposes a significant burden on patients’ families, healthcare systems, and socioeconomic resources due to repeated hospitalizations, severely impaired quality of life, and high mortality (4, 5).

Anxiety is a common comorbid psychological issue in heart failure patients, with approximately half affected by anxiety disorders (6). Heart failure may trigger anxiety through neurohormonal dysregulation, inflammatory responses, and autonomic nervous system imbalance. Anxiety not only significantly reduces patients’ quality of life and treatment adherence while decreasing physical activity, but may also accelerate cardiac function deterioration by activating neuroendocrine pathways and promoting inflammatory responses, forming a vicious cycle where heart failure and anxiety mutually reinforce each other (7, 8). Meanwhile, malnutrition, as one of the most common geriatric syndromes, is closely related to the occurrence and progression of cardiovascular diseases and patient prognosis (9, 10). Studies indicate that heart failure patients face a significantly elevated risk of malnutrition due to factors such as decreased appetite, impaired digestive and absorptive function, and metabolic disorders (11, 12). Malnutrition can further impact patients’ emotional health by impairing brain function and reducing physical reserves (13).

Notably, anxiety and malnutrition frequently coexist in heart failure patients and display synchronous correlations through multiple shared physiological mechanisms. Anxiety symptoms are correlated with chronic stress and poor nutritional reserves, and multiple shared physiological pathways may link these conditions together (14). Insufficient social support may indirectly increase nutritional risk by inducing anxiety and reducing appetite (15, 16). On the other hand, malnutrition may exacerbate anxiety symptoms by affecting neurotransmitter synthesis and brain function (17). This interaction of ‘heart failure progression-anxiety exacerbation-nutritional deterioration’ may establish multiple negative feedback loops, significantly increasing patients’ risk of adverse outcomes.

Although the relationships between heart failure and anxiety, and between heart failure and nutrition, have each received attention, systematic research incorporating heart failure, anxiety, and nutritional status within a unified framework remains scarce, particularly among elderly heart failure patients. Current research still lacks in-depth exploration of the association between anxiety and nutritional status in elderly heart failure patients. How these factors collectively influence heart failure severity (e.g., NYHA cardiac function classification, NT-proBNP levels) requires further elucidation.

Based on this, this study targeted elderly heart failure patients, aiming to: ① investigate the detection rate and independent influencing factors of anxiety symptoms in this population; ② explore the correlation between anxiety levels and nutritional condition. ③ analyze the interrelationship among anxiety, nutritional condition, and heart failure severity, to provide a basis for developing integrated management strategies combining psychological intervention and nutritional support in clinical practice.

## 2 Objects and methods

### 2.1 Objects

Using a cross-sectional design, 204 elderly heart failure patients hospitalized in the cardiovascular medicine department of The First Affiliated Hospital of Anhui University of Chinese Medicine from May to December 2025 were selected as the study subjects.

#### 2.1.1 Inclusion criteria

① Aged ≥65 years; ② meeting the diagnostic criteria outlined in the Chinese Guidelines for the Diagnosis and Management of Heart Failure 2024 (18); ③ classified as New York Heart Association Functional Classification (NYHA) class II to IV (19); ④ voluntarily participated in this study; ⑤ able to complete all Scale Assessments independently or with researcher assistance.

#### 2.1.2 Exclusion criteria

① Severe hepatic or renal insufficiency or advanced malignancies; ② severe cognitive dysfunction, persistent consciousness disturbance or diagnosed psychiatric disorders identified by professional geriatricians, and patients receiving regular anti-anxiety or antidepressant medication were excluded. All patients received unified cognitive assessment conducted by professional geriatricians immediately after admission before any scale (SAS, MNA-SF, etc.) measurement. The standardized screening was implemented based on the Clinical Dementia Rating (CDR) scale combined with the Mini-Cog test, and final judgments were supplemented by comprehensive clinical judgment of geriatricians. The assessment covered four fixed dimensions: time-place-person orientation, short-term memory, executive function and daily decision-making capacity. Clear unified diagnostic criteria for severe cognitive impairment were formulated, and all screening outcomes were recorded on identical standardized forms. To minimize subjective bias, all participating geriatricians completed unified standardized training and passed inter-rater consistency test with an agreement rate ≥90%. For patients with ambiguous cognitive performance, two geriatricians carried out independent separate evaluations to reach a consistent conclusion; ③Critical illness and end-stage disease; ④Concurrent participation in other clinical trials.

#### 2.1.3 Sample size estimation

We separately calculated adequate sample sizes for multiple linear regression and binary logistic regression per statistical standards. Among 18 collected indicators, only variables with significant univariate correlations entered multivariate models. Ten predictors were included in linear regression, meeting the minimum sample requirement of 100 subjects (20). For logistic regression, 99 anxiety events yielded an EPV of 19.8, far above the critical value of 10 to guarantee stable OR and CI estimation. A 15% potential attrition rate was preset to account for incomplete questionnaires and participant dropout. We recruited 236 eligible patients to reserve this buffer. After on-site sorting and double-data entry, 32 questionnaires were excluded due to missing core data, contradictory answers or voluntary participant withdrawal, leaving 204 valid samples. The actual attrition rate was 13.56%, lower than the pre-set 15%, confirming our initial sample inflation design was reasonable. The final valid sample of 204 satisfied the analytical needs of both regression models.

### 2.2 Research design and procedures

The research team consisted of cardiovascular disease specialists, professional nurses, and graduate students. All members received standardized training and participated in the study after passing the qualification assessment. To avoid assessment bias, Scale Assessment and clinical data collection for the same patient were independently performed by different researchers. Prior to the survey attending physicians confirmed that patients were clinically stable and cooperative. All assessments were completed within 3 days after admission, including baseline data collection, Scale Assessment, and laboratory indicator testing. All procedures adhered to standardized protocols.

### 2.3 Data collection and variable measurement

#### 2.3.1 Sociodemographic and clinical characteristics

Data were collected through structured questionnaires, physical examinations, and medical record reviews: (1) Sociodemographic information: gender, age, education level, marital status, living alone status, etc.; (2) Clinical features: years since heart failure diagnosis, NYHA cardiac function classification, medication regimen (including diuretics, beta-blockers, etc.), number of comorbid cardiovascular chronic conditions (including hypertension, coronary heart disease, arrhythmia, valvular heart disease, hyperlipidemia/dyslipidemia, etc.).

#### 2.3.2 Laboratory indicators

Fasting venous blood samples were collected in the morning within 3 days after hospital admission. Laboratory parameters were measured by professional technicians in the hospital laboratory department, including: N-terminal pro-brain natriuretic peptide (NT-proBNP), total protein (TP), Albumin (ALB), Albumin/Globulin ratio (A/G), Low-density lipoprotein cholesterol (LDL-C), High-density lipoprotein cholesterol (HDL-C).

#### 2.3.3 Psychological and functional assessment

All scales were assessed within 3 days after admission. If patients were unable to complete the scales independently, researchers conducted interviews item-by-item and recorded the responses accurately after obtaining consent from both the patients and their families.

All four questionnaires are officially published Chinese translated scales. We have contacted the original authors and Chinese distributors of these four measurement tools. According to their official statements, formal written authorization is generally waived for non-commercial academic research. In this manuscript, we have cited the original localization and validation papers for each scale and clearly stated that this study is carried out purely for non-profit research purposes, which complies with the official terms of use of all scales.

##### 2.3.3.1 Anxiety Status (Self-Rating Anxiety Scale, SAS)

Utilized the Self-Rating Anxiety Scale to assess patient anxiety status (21). The scale comprises 20 items, with the total score ranging from 25 to 100 after conversion. Patients were categorized as non-anxious with SAS < 50 and anxious with SAS ≥ 50. Scores of 50–59 indicated mild anxiety, 60–69 indicated moderate anxiety, and ≥70 indicated severe anxiety. Higher total scores corresponded to greater severity of anxiety. In the original Chinese localization research of SAS (22), the scale showed favorable criterion validity for evaluating anxiety symptoms among Chinese adults. The Cronbach’s *α* coefficient of SAS in our sample was 0.852.

##### 2.3.3.2 Nutritional Status (Mini Nutritional Assessment-Short Form, MNA-SF)

The Short-Form Mini Nutritional Assessment assessed patients’ nutritional condition with total scores ranging from 0 to 14. Scores of 0–7 indicated Malnutrition, 8–11 malnutrition risk, and 12–14 good nutritional status, where higher scores correspond to better nutritional status (23). The Chinese MNA-SF had a high correlation coefficient of *r* = 0.933 with the full MNA scale in the published validation study, which supported its reliable application in elderly nutritional screening (24). The Cronbach’s *α* coefficient of MNA-SF in this study was 0.716.

##### 2.3.3.3 Family Care Index (Family APGAR Index, APGAR)

The Family APGAR Index Questionnaire evaluated patients’ family support through 5 items, with total scores ranging from 0 to 10 (25, 26). Higher scores indicate better family care index. The Chinese Family APGAR Index had weighted Kappa >0.7 for the overall scale and most dimensions (adaptation dimension Kappa = 0.5519), and significant score differences before and after disease onset verified good construct validity (26). The Cronbach’s *α* coefficient of APGAR in our sample was 0.778.

##### 2.3.3.4 Quality of Life (Minnesota Living with Heart Failure Questionnaire, MLHFQ)

The Minnesota Living with Heart Failure Questionnaire was used to assess patients’ quality of life (27, 28). The scale comprises 21 items with a total score ranging from 0 to 105 points; lower total scores indicate better quality of life. The Chinese MLHFQ exhibited satisfactory psychometric properties: most domains had Cronbach’s *α* > 0.75 (only the economic limitation subscale had *α* = 0.50), stable two-week test–retest results (all *p* > 0.05), favorable responsiveness to clinical treatment, and strong structural correlation with NYHA classification (r = 0.64 ~ 0.70, *p* < 0.001) (28). The Cronbach’s *α* coefficient of MLHFQ calculated from our sample was 0.844.

### 2.4 Statistical methods

Data processing and analysis were performed using SPSS 27.0 statistical software, with the significance level set at *α* = 0.05.

#### 2.4.1 Descriptive statistics

Measurement data were initially tested for normality. Normally distributed data are presented as mean ± SD (X̄ ± S), while non-normally distributed data are expressed as median (M) and interquartile range (P_25_, P_75_). Categorical data are presented as frequency (*n*) and percentage (%).

#### 2.4.2 Univariate analysis

① Participants were categorized into the non-Anxiety group (SAS < 50) and the Anxiety group (SAS ≥ 50) based on anxiety status for univariate analysis of anxiety influencing factors. For intergroup comparisons, independent samples *t*-test was employed for normally distributed measurement data, Mann–Whitney *U* test for non-normally distributed measurement data, and *χ*^2^ test for count data. ② Univariate analysis of nutritional condition influencing factors was conducted using MNA-SF total score. Each study variable (sociodemographic information, clinical characteristics, laboratory indicators, APGAR total score, MLHFQ total score, etc.) was correlated with MNA-SF total score. Pearson correlation analysis was applied to normally distributed measurement data, while Spearman’s rank correlation analysis was used for non-normally distributed measurement data and categorical variables. Variables demonstrating statistically significant associations with nutritional condition (*p* < 0.05) were identified.

#### 2.4.3 Multivariate analysis

① Binary logistic regression was used to screen independent risk factors for anxiety (dependent variable: 0 = non-anxiety, 1 = anxiety). Prior to regression modeling, the Box-Tidwell test was performed to verify the linearity assumption between each continuous predictor and the logit of anxiety. We generated interaction terms of each continuous variable multiplied by its natural logarithm and incorporated these interaction terms into the full multivariate model with all included covariates for testing. The *p* values of the three interaction terms were 0.305 (LDL_inter), 0.151 (HDL_inter) and 0.630 (MNASF_inter), all greater than 0.05, which confirmed the linear association. If nonlinearity had been observed, restricted cubic splines or clinically based categorical grouping would have been adopted for variable transformation. Two coding strategies were applied to independent variables: NYHA classification, living alone status and NT-proBNP were dichotomized by clinical criteria; NYHA class II = 0, class III–IV = 1; living alone (yes = 1, no = 0); NT-proBNP ≥ 800 ng/L was coded as 1, NT-proBNP < 800 ng/L as 0. Biochemical indices (HDL-C, LDL-C, TP, ALB), MNA-SF, APGAR and MLHFQ scores were input as original continuous values without grouping. All variable assignment standards are shown in Supplementary Table 2. Stepwise regression with entry criterion 0.05 and removal criterion 0.10 was performed to calculate *B*, SE, Wald *χ*^2^, *p*, OR and 95% CI.

② Multiple stepwise linear regression was conducted to identify independent predictors of MNA-SF nutritional score (continuous dependent variable). All variables with *p* < 0.05 in univariate analysis were entered as predictors. Collinearity diagnosis based on tolerance and variance inflation factor (VIF) was implemented; variables with VIF > 10 were regarded as severe multicollinear and excluded to ensure stable regression coefficients. All predictors retained in the final regression model had VIF values below 5, without obvious collinearity interference. The output indicators included unstandardized *B*, SE, standardized *β*, *t*-value and *p*, with model fit statistics (*F*, adjusted *R*^2^) reported.

③ A triangular conceptual framework (Supplementary Figure S1) was plotted to distinguish direct pairwise correlations and indirect mediated associations among heart failure severity, nutritional status and anxiety.

## 3 Results

### 3.1 Comparison of clinical data between the two groups

Statistically significant differences were observed between the non-Anxiety group and the Anxiety group in NYHA cardiac function classification, number of comorbid chronic cardiovascular diseases, living alone status, NT-proBNP, HDL-C, LDL-C, TP, ALB, MNA-SF total score, APGAR total score, and MLHFQ total score (all *p* < 0.05); No statistically significant differences were found in gender, age, education level, marital status, duration of heart failure diagnosis, number of medication types, or A/G (all *p* > 0.05), as detailed in Supplementary Table 1.

In addition, the sample was unevenly distributed across age (65–79: *n* = 86; ≥80: *n* = 118) and NYHA subgroups (Class II: *n* = 38; Class III: *n* = 113; Class IV: *n* = 53). All multivariate models were fitted on the total sample with age and NYHA grade adjusted as covariates, rather than stratified subgroup analyses.

### 3.2 Binary logistic regression analysis of factors influencing anxiety in elderly heart failure patients

Binary logistic regression analysis revealed that NYHA cardiac function classification, NT-proBNP, and LDL-C were independent risk factors for anxiety in elderly heart failure patients, whereas HDL-C and MNA-SF total score were independent protective factors (all *p* < 0.05). Patients with NT-proBNP ≥ 800 ng/L had an odds ratio of 12.177 for anxiety relative to patients with NT-proBNP < 800 ng/L. For each one-unit increment in LDL-C, the odds of anxiety increased (OR = 2.029); each one-unit elevation in HDL-C and MNA-SF was associated with reduced anxiety odds (OR = 0.264 and OR = 0.434, respectively). Additionally, the number of comorbid cardiovascular chronic diseases, living alone status, TP, ALB, APGAR total score, and MLHFQ total score showed no significant independent influence on anxiety occurrence (all *p* > 0.05), as shown in Supplementary Table 3.

### 3.3 Correlation analysis between nutritional status and clinical indicators in elderly heart failure patients

Spearman’s rank correlation analysis revealed that the MNA-SF total score in elderly heart failure patients showed significant negative correlations with NYHA cardiac function classification, NT-proBNP, LDL-C, MLHFQ total score, and SAS total score (rs = −0.163 to −0.832, all *p* < 0.05), with the strongest correlation observed with the SAS total score (rs = −0.832, *p* < 0.01). Demonstrated significant positive correlations with HDL-C, TP, ALB, and APGAR total score (rs = 0.139–0.402, all *p* < 0.05). Furthermore, nutritional condition exhibited moderate-to-high correlations with quality of life and family care index, suggesting close interrelationships among nutritional status, psychological factors, quality of life, and family support. These findings indicate that better nutritional status in elderly heart failure patients associates with improved cardiac function, favorable lipid metabolism, reduced anxiety levels, higher family care index, and enhanced quality of life. Notably, the association between nutritional status and anxiety severity was most significant, as detailed in Supplementary Table 4.

### 3.4 Multiple linear regression analysis of factors influencing nutritional status in elderly heart failure patients

Using the MNA-SF total score as the dependent variable, variables demonstrating statistically significant associations with nutritional status (*p* < 0.05) in Supplementary Table 4 NYHA cardiac function classification, number of comorbid cardiovascular chronic diseases, NT-proBNP, HDL-C, LDL-C, TP, ALB, APGAR total score, MLHFQ total score, and SAS total score—were included as independent variables. Assignments followed above. Multiple stepwise linear regression analysis identified independent factors influencing nutritional status in elderly heart failure patients, with a significance level of *α* = 0.05, an inclusion criterion of 0.05, and an exclusion criterion of 0.10.

The results showed that this multiple stepwise linear regression model demonstrated good overall fit (*F* = 47.726, *p* < 0.001, adjusted *R*^2^ = 0.698), indicating that the included independent variables collectively explained 69.8% of the variation in nutritional condition among elderly heart failure patients. Among the variables that ultimately entered the model, NT-proBNP levels, MLHFQ total score, and SAS total score were identified as independent risk factors for nutritional condition in elderly heart failure patients (all *p* < 0.05), while APGAR total score was an independent protective factor (*p* < 0.05). Specifically, higher NT-proBNP levels and per one-point increase in MLHFQ and SAS total scores were associated with worse nutritional status, whereas higher APGAR total scores were associated with better nutritional status. NYHA cardiac function classification, number of comorbid cardiovascular chronic diseases, HDL-C, LDL-C, TP, and ALB showed no statistically significant impact on nutritional condition (all *p* > 0.05), as presented in Supplementary Table 5. Collinearity diagnosis showed all included independent variables had VIF < 5, and the high correlation between SAS and MLHFQ did not cause serious multicollinearity bias to the regression coefficients and standard errors in this sample.

## 4 Discussion

This study enrolled 204 elderly heart failure patients and investigated factors influencing anxiety along with correlations between nutritional condition, anxiety, and heart failure severity through multidimensional analysis. The findings revealed an anxiety prevalence of 48.5%, with NYHA cardiac function classification and NT-proBNP identified as independent risk factors for anxiety, while nutritional condition (MNA-SF total score) emerged as a core independent protective factor. Variables related to social support demonstrated only univariate correlations with anxiety and did not emerge as independent influencing factors. This study elucidates the underlying mechanisms by examining shared pathophysiological pathways among these three elements, the role of social support, and the bridging role of nutrition, thereby providing references for integrated clinical management.

### 4.1 Correlation between heart failure severity and anxiety

This study demonstrated significant positive correlations between total anxiety scores and both NYHA cardiac function classification (rs = 0.443, *p* < 0.01) and NT-proBNP levels (rs = 0.312, *p* < 0.01). The proportion of NT-proBNP-positive patients was significantly higher in the Anxiety group (64.1%) compared to the non-Anxiety group (35.9%), confirming a close association between cardiac function deterioration and anxiety. These findings align with multiple previous studies establishing the relationship between worsening cardiac function and anxiety (29–31). Logistic regression analysis revealed that patients with NYHA class III-IV had a 7.826-fold increased risk of anxiety compared to class II patients, while NT-proBNP-positive patients exhibited a 12.177-fold higher anxiety risk than negative counterparts. Both were identified as independent risk factors for anxiety, with NT-proBNP demonstrating stronger predictive power—a finding consistent with previous studies (32, 33).

The bidirectional association between these factors stems from shared pathophysiological pathways involving neuroendocrine activation, chronic inflammatory response, and autonomic nervous system imbalance: Heart failure-induced physical symptoms (e.g., dyspnea and fatigue) restrict daily activities, compounded by medical burdens from recurrent hospitalizations and prognostic uncertainty, thereby exacerbating psychological stress and triggering anxiety (6); Anxiety activates the sympathetic nervous system and hypothalamic–pituitary–adrenal (HPA) axis, which both increases cardiac load and exacerbates myocardial remodeling through chronic inflammatory responses, reducing cardiac functional reserve (34), forming a vicious cycle of ‘heart failure deterioration-anxiety aggravation’.

This study also found that the number of comorbid cardiovascular chronic diseases was significantly higher in the Anxiety group than in the non-Anxiety group, consistent with previous findings that over 75% of elderly heart failure patients have three or more chronic comorbidities and approximately 50% have five or more (35), but this variable was not included in the multivariate model, potentially due to collinearity with NYHA cardiac function classification and NT-proBNP (as multimorbidity clinically often coexists with worsening cardiac function and elevated NT-proBNP, demonstrating a natural association). Heart failure severity explained partial variance, and confounding factors like elderly patients’ physical tolerance and disease management capacity diluted its independent effect. Multimorbidity was correlated with more severe symptoms and heavier disease management load, and showed a synchronous association with higher anxiety levels (36). Therefore, for such elderly heart failure patients with multimorbidity, clinical practice should integrate psychological health assessment into routine diagnostic workflows, combining cardiovascular disease management and psychological intervention through multidisciplinary collaboration (37), thereby alleviating the cumulative effects of comorbidities and reducing the risk of anxiety development.

Binary logistic regression analysis revealed that NYHA cardiac function classification, NT-proBNP, and LDL-C were independent risk factors for anxiety (all *p* < 0.05), whereas HDL-C and MNA-SF total score were independent protective factors (all *p* < 0.05). In addition, LDL-C and HDL showed independent associations with anxiety in our model, a finding inconsistent with some prior literature. The discrepancy mainly lies in study populations and confounding adjustment: most earlier work enrolled mixed-age cardiovascular cohorts and failed to simultaneously control for cardiac function, nutrition and family support. Elderly heart failure patients suffer from persistent visceral congestion and chronic inflammation, which amplifies the lipid-emotion correlation after comprehensive covariate correction, which supports the credibility of our results. Patients with NT-proBNP ≥ 800 ng/L had an odds ratio of 12.177 for anxiety relative to patients with NT-proBNP < 800 ng/L. Each unit rise in LDL-C elevated anxiety odds (OR = 2.029); each unit increase in HDL-C and MNA-SF corresponded to reduced anxiety risk (OR = 0.264 and OR = 0.434, respectively).

We further stratified the specific biological pathways linking dyslipidaemia and anxiety in elderly heart failure patients. Excess LDL-C promotes systemic low-grade inflammation via lipid peroxidation and macrophage foam cell formation; elevated inflammatory cytokines further activate the HPA axis and sympathetic tone, aggravating anxious mood (38). In contrast, insufficient HDL loses its anti-inflammatory and blood–brain barrier protective effects, reducing the clearance of neurotoxic lipid metabolites and impairing central neurotransmitter balance (39). For aged heart failure individuals, age-related hepatic lipid clearance decline, intestinal malabsorption caused by long-term visceral venous congestion, and insufficient protein and lipid intake jointly exacerbate dyslipidaemia, forming a unique metabolic disturbance characteristic of this population (40). Moreover, abnormal lipid levels also interact with nutritional status: inadequate dietary lipid intake lowers HDL and elevates inflammatory stress simultaneously, acting as an intermediate mediator connecting poor nutrition and increased anxiety risk within the heart failure-nutrition-anxiety triangular interaction.

We systematically compared anxiety prevalence data from domestic and international cohorts focusing on elderly heart failure patients. Domestic single-centre cross-sectional studies reported anxiety prevalence ranging from 23 to 74% among hospitalised elderly heart failure patients, while European and American studies mostly recorded a lower range of 35–42% (6, 7). Differences in psychological assessment tools and regional elderly care systems are common explanations for such inconsistent prevalence rates across studies. Beyond these widely discussed factors, the TCM-integrated features of our study sample also deserve consideration. Some patients admitted to our ward received standard TCM emotional intervention and specialized dietary management during hospitalization. These ward-specific auxiliary care measures are rarely available in Western cardiovascular wards overseas and may subtly alter patients’ SAS scores, representing an extra factor contributing to the inter-study discrepancy in anxiety detection rates. Most overseas research adopted brief scales such as GAD-7 or HADS, whereas this study applied the full SAS scale covering somatic, emotional and sleep-related anxiety symptoms, which partly explains the 48.5% anxiety prevalence observed in our sample.

Therefore, for elderly heart failure patients complicated with multiple chronic diseases, combined cardiovascular and psychological assessment should be standardised to mitigate the cumulative adverse impacts of multimorbidity.

### 4.2 Buffering effect of social support on anxiety

Social support serves as a crucial protective factor for the psychological well-being of elderly heart failure patients, buffering negative stress induced by the disease and mitigating the development of anxiety (41). Univariate analysis in this study demonstrated a significant association between living alone and anxiety. The non-Anxiety group exhibited significantly higher APGAR total scores than the Anxiety group, corroborating social support’s buffering effect on negative emotions—living alone amplifies such emotions through deficient caregiving and emotional companionship (42); A high family care index can reduce disease fluctuations through practical care provision and offer emotional outlets via affective support (43). However, neither living alone nor the family care index entered the multivariate regression model for anxiety. Higher APGAR family support score was independently positively correlated with better nutritional status among elderly heart failure patients, indicating that adequate family support was associated with improved nutritional condition of hospitalized elderly heart failure individuals.

However, the correlation between family support and anxiety symptoms only existed in univariate analysis. After adjusting for cardiac function grading, laboratory biochemical indicators and quality-of-life as well as anxiety scale scores, family support lost independent predictive significance for anxiety. We only objectively report these two separate correlational results derived from different regression models, and no inference about indirect mediating pathway linking family support, nutritional status and anxiety is proposed. Clinically, it is essential to prioritize social support assessment and establish diversified support systems for individuals living alone or with low family care index to fully leverage their buffering effect on patients’ mood.

### 4.3 The critical impact of nutritional status on the relationship between heart failure and anxiety

As illustrated in Supplementary Figure S1, we put forward a theoretical speculation of the heart failure-nutrition-anxiety triangular synchronous correlation model based on our correlation data. It should be clearly emphasized that this cross-sectional study only captures synchronous associations of indicators and cannot distinguish causal sequences; this triangular interaction model is merely an inferential hypothesis and needs further longitudinal verification. We systematically sorted the interrelated direct and indirect biological pathways supported by existing evidence, and quantitatively compared the strength of pairwise correlations among the three core indicators. First, we quantified the magnitude of direct pairwise correlations using Spearman correlation coefficients. The strongest direct correlation existed between nutritional status and anxiety (rs = −0.832). The moderate correlation between heart failure severity and anxiety ranked second (rs = 0.351), whereas the association between heart failure severity and nutritional status was relatively weak (rs = −0.283). Solid bidirectional lines in Supplementary Figure S1 represent these direct synchronous correlations between each pair of indicators. Second, the dotted lines in the figure indicate indirect associations mediated by shared neuroendocrine, inflammatory and intestinal metabolic pathways, which clearly distinguishes direct and indirect interactions. Three coherent hierarchical mechanisms supported by previous evidence are summarized as follows:

Advanced heart failure leads to visceral venous congestion and intestinal mucosal ischemia-hypoxia, which triggers persistent low-grade systemic inflammation and impairs intestinal nutrient absorption, jointly correlating with poor nutritional status (44, 45). Elevated NT-proBNP, as a marker of severe cardiac dysfunction, is synchronously linked to nutritional deterioration. Insufficient intake of protein substrates restricts the synthesis of serotonin and other mood-regulating neurotransmitters; hypoproteinemia impairs central nervous regulation, which is closely correlated with elevated anxiety symptoms (46, 47). Sustained anxiety activates the sympathetic nervous system and hypothalamic–pituitary–adrenal (HPA) axis to raise circulating cortisol (34). High cortisol levels suppress ghrelin secretion and reduce appetite intake, while chronic inflammatory activation increases cardiac afterload, forming simultaneous correlational trends of aggravated cardiac dysfunction and worse nutritional indicators.

Cardiac dysfunction, malnutrition and anxiety are interrelated through shared neuroendocrine, inflammatory and intestinal metabolic pathways, forming a set of synchronized negative correlations rather than confirmed bidirectional causal loops.

This visualized triangular framework clarifies the hierarchical logic of variable interactions, rather than listing three independent pathways separately. This triangular correlational pattern provides a theoretical reference for clinical integrated management. In routine geriatric heart failure care, parallel screening of psychological state and nutritional risk using SAS and MNA-SF is worthy of recommendation. Targeted protein supplementation and mental care may help improve the overall clinical profile of patients.

### 4.4 Study limitations and future research directions

This study has several limitations: ① the sample was recruited from a single center, which may limit generalizability; the wide 95% CIs of some logistic regression estimates reflected limited statistical precision due to the sample size and multiple independent variables included. ② We did not record indicators including fluid balance and edema, nor long-term prognostic outcomes such as readmission and all-cause mortality. ③ As a cross-sectional design, this work can not verify causal directional relationships between any measured variables, only synchronous correlations can be identified. ④ We excluded patients with active psychiatric disorders and individuals receiving regular antidepressant or anxiolytic therapy to eliminate major confounding bias related to psychiatric medication. This exclusion screens out patients whose anxiety symptoms are controlled by medication, which may artificially lower the observed anxiety prevalence. Accordingly, the 48.5% anxiety prevalence reported in this study cannot fully reflect the overall anxiety burden of elderly heart failure patients in real clinical settings. In addition, occasional short-term sedative use for insomnia was not systematically recorded in medical charts, which may bring minor residual confounding. Only a tiny share of participants reported intermittent sleep aids during ward rounds, so the overall bias magnitude is modest. Future research should collect complete records of all psychotropic agents and incorporate medication data as adjustment covariates in multivariate regression models. ⑤ The sample showed unbalanced distribution across age and NYHA functional subgroups, which restricts us from conducting stratified subgroup analyses.

Future research could focus on four aspects: ① conduct multicenter prospective cohort studies to expand sample size, improve statistical precision, and clarify potential causal pathways; ② design randomized controlled trials to compare the efficacy of combined psychological and nutritional intervention regimens; ③ add assessment indicators such as coping styles and community social support to refine the multivariate model; ④ screen and validate serum biomarkers linked to anxiety risk in elderly heart failure patients. ⑤ enroll both patients with and without long-term antidepressant/anxiolytic treatment to avoid underestimation of anxiety prevalence and obtain a more accurate estimate of the real-world anxiety burden; ⑥ future multi-center large-cohort studies will adopt stratified sampling with predetermined sample ratios for each subgroup to achieve sufficient statistical power and further explore subgroup differences.

## 5 Conclusion

This study found that the prevalence of anxiety among elderly heart failure patients reached 48.5%, with heart failure severity being the core risk factor for anxiety, while good nutritional status served as a significant protective factor. Clinical practice should incorporate psychological and nutritional assessments into routine diagnostics and treatment, establishing an integrated management model. This study provides evidence-based support for the association among these three factors; subsequent multicenter prospective studies are required to verify causality and optimize clinical pathways.

## Data Availability

The original contributions presented in the study are included in the article/Supplementary material, further inquiries can be directed to the corresponding authors.
