# Supplementary material for: Study on influencing factors of anxiety symptoms and its association with nutritional condition in elderly heart failure patients
Source: Front Nutr. 2026 Aug 12;13:1861582. doi: 10.3389/fnut.2026.1861582 (PMC13506413; doi:10.3389/fnut.2026.1861582)
Supplement: Supplementary file 1 [file Supplementary_file_1.DOCX]

Supplementary Material

# Supplementary Tables

**Supplementary Table 1 General Information of Patients in the Non-Anxiety and Anxiety Groups**

| **Variables** | **SAS Score** | | **Total (n)** | **Statistical Value** | ***P-value*** | **Effect Size** |
| --- | --- | --- | --- | --- | --- | --- |
|  | **<50 points (non-Anxiety group) (n=105)** | **≥50 points (Anxiety group) (n=99)** |  |  |  |  |
| Gender |  |  |  |  |  |  |
| Male | 55(57.3) | 41(42.7) | 96 | 2.460① | 0.117 | 0.110① |
| Female | 50(46.3) | 58(53.7) | 108 |  |  |  |
| Age | 80.44±7.438 | 79.25±6.946 | - | 1.175② | 0.241 | 0.165③ |
| Education Level |  |  |  |  |  |  |
| Junior high school and below | 59(54.6) | 49(45.4) | 108 | -0.489③ | 0.625 | 0.118② |
| Technical secondary school and high school | 23(41.8) | 32(58.2) | 55 |  |  |  |
| College degree and above | 23(56.1) | 18(43.9) | 41 |  |  |  |
| Marital Status |  |  |  |  |  |  |
| Married | 89(50.9) | 86(49.1) | 175 | -0.430③ | 0.667 | 0.030① |
| Widowed | 16(55.2) | 13(44.8) | 29 |  |  |  |
| Time since heart failure diagnosis (years) | 3.2029±2.81655 | 3.8737±2.99818 | - | -1.648② | 0.101 | 0.231③ |
| NYHA cardiac function classification |  |  |  | -4.825③ | < 0.001 |  |
| Class II | 33(86.8) | 5(13.2) | 38 |  |  | 0.339① |
| Class III to IV | 72(43.4) | 94(56.6) | 166 |  |  |  |
| Number of medications | 4.29±1.498 | 4.39±1.490 | - | -0.517② | 0.606 | 0.072③ |
| Number of cardiovascular chronic diseases Number of comorbidities | 2.42±1.543 | 3.76±1.922 | - | -5.464② | < 0.001 | 0.770③ |
| Living alone status |  |  |  |  |  |  |
| No | 69(45.7) | 82(54.3) | 151 | 7.761① | 0.005 | 0.195① |
| Yes | 36(67.9) | 17(32.1) | 53 |  |  |  |
| NT-proBNP |  |  |  |  |  |  |
| NT-proBNP Positive | 46(35.9) | 82(64.1) | 128 | 33.188① | < 0.001 | 0.403① |
| NT-proBNP Negative | 59(77.6) | 17(22.4) | 76 |  |  |  |
| HDL-C | 1.19(1.05~1.34) | 1.04(0.845~1.365) | - | -2.603③ | 0.009 | 0.182④ |
| LDL-C | 2.3255±0.72808 | 2.7246±0.89597 | - | -3.491② | < 0.001 | 0.490③ |
| TP | 67.2146±6.72686 | 63.6384±9.15683 | - | 3.164② | 0.002 | 0.447③ |
| ALB | 40.2(36.5~42.6) | 37.2(34.45~39.95) | - | -4.021③ | < 0.001 | 0.321③ |
| A/G | 1.43(1.23~1.61) | 1.34(1.18~1.61) | - | -1.170③ | 0.242 | 0.082④ |
| Malnutrition present | 4(6.8) | 55(93.2) | 59 |  |  | 0.690② |
| At risk of malnutrition | 42(50.0) | 42(50.0) | 84 |  |  |  |
| Normal nutritional status | 59(96.7) | 2(3.3) | 61 |  |  |  |
| MNA-SF total score | 11.5048±2.03396 | 6.7778±2.65559 | - | 14.212② | < 0.001 | 2.006③ |
| APGAR total score | 7.67±2.239 | 5.99±2.573 | - | 4.973② | < 0.001 | 0.697③ |
| Physical domain | 22.14±6.782 | 28.08±7.663 | - | -5.868② | < 0.001 |  |
| Emotional domain | 11.30±4.147 | 17.20±4.980 | - | -9.212② | < 0.001 |  |
| Other domains | 21(17~23) | 24(20~27) | - | -4.802③ | < 0.001 |  |
| MLHFQ total score | 54.01±13.586 | 69.04±13.828 | - | -7.829② | < 0.001 | 1.097③ |

Note: Normally distributed measurement data are expressed as mean ± standard deviation; Non-normally distributed measurement data are expressed as M(P_25_, P_75_).

① Chi-square value; ② t-value; ③ Z-value.

Effect size indicators: φ for 2×2 chi-square test (①); Cramer’s V for multi-category chi-square test (②); Cohen’s d for independent samples t-test (③);$\text{r}\text{=}\frac{\text{|}\text{z}\text{|}}{\sqrt{\text{N}}}$ for Mann-Whitney U test (④).

Medication types primarily included: diuretics, beta-blockers, statins, calcium channel blockers, digitalis preparations, etc.

Comorbid cardiovascular chronic diseases primarily included: hypertension, coronary heart disease, arrhythmias, valvular heart disease, hyperlipidemia/dyslipidemia, etc.

NT-proBNP is N-terminal pro-brain natriuretic peptide (NT-proBNP > 800 ng/L was considered positive; NT-proBNP ≤ 800 ng/L was considered negative).

HDL-C denotes High-density lipoprotein cholesterol; LDL-C represents low-density lipoprotein cholesterol; TP signifies total protein; ALB indicates Albumin; and A/G refers to the Albumin/Globulin ratio.

| **Supplementary Table 2 Assignment Methods** | |
| --- | --- |
| **Variables** | **Assignment Methods** |
| NYHA cardiac function classification | Grade II = 0, Grade III-IV = 1 |
| Combined cardiovascular diseases Number of chronic diseases | Actual value input |
| Living alone status | No = 0, Yes = 1 |
| NT-proBNP | NT-proBNP≥800ng/L=1 NT-proBNP＜800ng/L=0 |
| HDL-C | Actual value input |
| LDL-C | Actual value input |
| TP | Actual value input |
| ALB | Actual value input |
| MNA-SF total score | Actual value input |
| APGAR total score | Actual value input |
| MLHFQ total score | Actual value input |

**Supplementary Table 3 Results of binary logistic regression analysis of factors influencing anxiety symptoms in elderly heart failure patients**

| **Independent variable** | ***B*** | ***SE*** | ***Wald χ² value*** | ***P-value*** | ***OR*** | ***95%CI*** |
| --- | --- | --- | --- | --- | --- | --- |
| NYHA Cardiac function classification | 2.057 | 0.778 | 6.99 | 0.008 | 7.826 | 1.703~35.972 |
| Combined cardiovascular diseases Number of chronic diseases | 0.269 | 0.139 | 3.712 | 0.054 | 1.308 | 0.995~1.719 |
| Living alone status | -0.737 | 0.619 | 1.415 | 0.234 | 0.479 | 0.142~1.612 |
| NT-proBNP | 2.5 | 0.689 | 13.169 | <0.001 | 12.177 | 3.157~46.97 |
| HDL-C | -1.331 | 0.634 | 4.412 | 0.036 | 0.264 | 0.076~0.915 |
| LDL-C | 0.707 | 0.358 | 3.904 | 0.048 | 2.029 | 1.006~4.092 |
| TP | -0.016 | 0.038 | 0.177 | 0.674 | 0.984 | 0.913~1.061 |
| ALB | 0.011 | 0.051 | 0.051 | 0.821 | 1.011 | 0.916~1.117 |
| APGAR total score | 0.045 | 0.128 | 0.123 | 0.725 | 1.046 | 0.814~1.344 |
| MNA-SF total score | -0.835 | 0.174 | 23.02 | <0.001 | 0.434 | 0.308~0.61 |
| MLHFQ total score | 0.025 | 0.019 | 1.681 | 0.195 | 1.025 | 0.987~1.064 |

**Supplementary Table 4 Correlation Between Nutritional Status in Elderly Heart Failure Patients and NYHA Cardiac Function Classification, Family Care Index Total Score, Quality of Life Assessment Total Score, and Related Factors**

|  | **MNA-SF Total Score** | **NYHA Cardiac Function Classification** | **Number of Comorbid Cardiovascular Chronic Diseases** | **NT-pro**  **BNP** | **HDL-C** | **LDL-C** | **TP** | **ALB** | **APGAR Total Score** | **MLHFQ Total Score** | **SAS Total Score** |
| --- | --- | --- | --- | --- | --- | --- | --- | --- | --- | --- | --- |
| **MNA-SF Total Score** | 1 |  |  |  |  |  |  |  |  |  |  |
| New York Heart Association (NYHA) classification | -0.283**  (-0.408 ~ -0.148) |  |  |  |  |  |  |  |  |  |  |
| **Number of Comorbid Cardiovascular Chronic Diseases** | -0.324**  (-0.445 ~ -0.191 | 0.248**  (0.111 ~ 0.376 |  |  |  |  |  |  |  |  |  |
| **NT-proBNP** | -0.334**  (-0.454 ~ -0.202 | 0.126  (-0.016 ~ 0.263 | 0.266**  (0.130 ~ 0.393 |  |  |  |  |  |  |  |  |
| **HDL-C** | 0.139*  (-0.002 ~ 0.275 | -0.075  (-0.214 ~ 0.067 | -0.014  (-0.155 ~ 0.128 | 0.037  (-0.105 ~ 0.178 |  |  |  |  |  |  |  |
| **LDL-C** | -0.163*  (-0.298 ~ -0.022 | 0.115  (-0.027 ~ 0.253 | 0.199**  (0.059 ~ 0.331 | 0.103  (-0.039 ~ 0.242 | 0.088  (-0.055 ~ 0.227 |  |  |  |  |  |  |
| **TP** | 0.276**  (0.140 ~ 0.401 | -0.092  (-0.230 ~ 0.050 | -0.075  (-0.214 ~ 0.067 | 0.007  (-0.135 ~ 0.148 | 0.098  (-0.044 ~ 0.236 | -0.011  (-0.153 ~ 0.131 |  |  |  |  |  |
| **ALB** | 0.258**  (0.121 ~ 0.385 | -0.103  (-0.241 ~ 0.039 | -0.138*  (-0.274 ~ 0.003 | -0.144*  (-0.279 ~ -0.002 | 0.047  (-0.095 ~ 0.187 | 0.034  (-0.109 ~ 0.175 | 0.364**  (0.235 ~ 0.481 |  |  |  |  |
| **APGAR Total Score** | 0.402**  (0.277 ~ 0.514 | -0.102  (-0.240 ~ 0.040 | -0.077  (-0.216 ~ 0.066 | -0.081  (-0.220 ~ 0.061 | 0.170*  (0.030 ~ 0.304 | -0.157*  (-0.292 ~ -0.015 | 0.169*  (0.029 ~ 0.304 | 0.044  (-0.098 ~ 0.184 |  |  |  |
| **MLHFQ Total Score** | -0.620**  (-0.700 ~ -0.525 | 0.314**  (0.181 ~ 0.436 | 0.145*  (0.004 ~ 0.281 | 0.175*  (0.035 ~ 0.309 | -0.142*  (-0.278 ~ 0.000 | 0.052  (-0.090 ~ 0.192 | -0.151*  (-0.286 ~ -0.010 | -0.201**  (-0.333 ~ -0.061 | -0.307**  (-0.430 ~ -0.173 |  |  |
| **SAS Total Score** | -0.832**  (-0.871 ~ -0.782 | 0.351**  (0.221 ~ 0.469 | 0.364**  (0.235 ~ 0.481 | 0.312**  (0.178 ~ 0.434 | -0.151*  (-0.287 ~ -0.010 | 0.246**  (0.108 ~ 0.374 | -0.227**  (-0.357 ~ -0.089 | -0.268**  (-0.394 ~ -0.131 | -0.409**  (-0.521 ~ -0.284 | 0.608**  (0.511 ~ 0.690 | 1.000 |

Note:* denotes *P*<0.05, ** denotes *P*<0.01.
These correlation analysis results provide a foundation and assurance for subsequent research on influencing factors.The 95% confidence intervals of all Spearman correlation coefficients were calculated using Fisher r-to-z transformation with n=204.

**Supplementary Table 5 Results of multiple linear regression analysis on factors influencing nutritional risk in elderly heart failure patients**

| **Independent variable** | ***B*** | ***SE*** | ***β*** | ***t-value*** | ***P-value*** |
| --- | --- | --- | --- | --- | --- |
| NYHA cardiac function classification | 0.159 | 0.359 | 0.019 | 0.443 | 0.659 |
| Number of Comorbid Cardiovascular Chronic Diseases | -0.069 | 0.077 | -0.038 | -0.892 | 0.374 |
| NT-proBNP | -0.602 | 0.288 | -0.087 | -2.089 | 0.038 |
| HDL-C | -0.290 | 0.322 | -0.036 | -0.902 | 0.368 |
| LDL-C | 0.093 | 0.163 | 0.023 | 0.574 | 0.567 |
| TP | 0.024 | 0.017 | 0.058 | 1.435 | 0.153 |
| ALB | 0.025 | 0.022 | 0.045 | 1.128 | 0.261 |
| APGAR total score | 0.121 | 0.059 | 0.093 | 2.069 | 0.040 |
| MLHFQ total score | -0.036 | 0.011 | -0.169 | -3.375 | <0.001 |
| SAS total score | -0.173 | 0.016 | -0.632 | -10.635 | <0.001 |

Collinearity diagnostics (VIF) were performed for all independent variables in this multiple linear regression model, and all VIF < 5.

**Supplementary Figure S1 Conceptual framework of triangular interactions among heart failure severity, nutritional status and anxiety in elderly hospitalized heart failure patients。**

Solid bidirectional lines represent direct pairwise correlations with corresponding Spearman’s correlation coefficients (rs) marked above each line; line thickness is proportional to the absolute value of rs. Dotted bidirectional lines represent indirect associations mediated by chronic inflammation, lipid metabolism disorders (LDL-C/HDL-C) and HPA neuroendocrine activation. ***P*<0.01.
